# Targeting oncogenic Notch signaling with SERCA inhibitors

**DOI:** 10.1186/s13045-020-01015-9

**Published:** 2021-01-06

**Authors:** Luca Pagliaro, Matteo Marchesini, Giovanni Roti

**Affiliations:** Department of Medicine and Surgery, University of Parma, 43126 Parma, Italy

**Keywords:** SERCA, T cell acute lymphoblastic leukemia, Thapsigargin, Notch signaling, NOTCH1, CAD204520, T-ALL

## Abstract

P-type ATPase inhibitors are among the most successful and widely prescribed therapeutics in modern pharmacology. Clinical transition has been safely achieved for H^+^/K^+^ ATPase inhibitors such as omeprazole and Na^+^/K^+^-ATPase inhibitors like digoxin. However, this is more challenging for Ca^2+^-ATPase modulators due to the physiological role of Ca^2+^ in cardiac dynamics. Over the past two decades, sarco-endoplasmic reticulum Ca^2+^-ATPase (SERCA) modulators have been studied as potential chemotherapy agents because of their Ca^2+^-mediated pan-cancer lethal effects. Instead, recent evidence suggests that SERCA inhibition suppresses oncogenic Notch1 signaling emerging as an alternative to γ-secretase modulators that showed limited clinical activity due to severe side effects. In this review, we focus on how SERCA inhibitors alter Notch1 signaling and show that Notch on-target-mediated antileukemia properties of these molecules can be achieved without causing overt Ca^2+^ cellular overload.

## Background

NOTCH receptors are transmembrane cell-surface proteins that control cell to cell communication, embryogenesis, and tissue commitment [1]. In mammals, there are four NOTCH isoforms (I-IV) that share a similar basic structure organized in modules, including an extracellular domain (NECD) at the N‐terminus, a transmembrane domain (NTM), and a NOTCH intracellular domain (NICD) at the C‐terminus. After translation, pro-NOTCH proteins are proteolytically cleaved in the endoplasmic reticulum (ER)/Golgi compartment by a furin-like protease [2]. This cleavage (S1) releases non-covalent heterodimers that, once translocated on the surface of the cells [3, 4], are activated by the binding with ligands (Delta-like 1, 3, 4 and Jagged 1, 2) expressed on the neighboring cells [5]. This interaction generates a NOTCH conformational change that exposes site 2 (S2) to the proteolytic activity of a disintegrin and metalloprotease (ADAM-10 or TACE/ADAM-17). The resulting short-lived protein fragments are substrates of the γ**-**secretase complex (S3) that releases NICD to the cytoplasm [6, 7]. Then, NICD translocates into the nucleus, associates with the co-transcription factor of the Mastermind-like gene family (MAML) [8], and complex with the p300/CBF‐associated (PCAF) co-activators to bind the DNA-binding factor recombination signal binding protein for immunoglobulin kappa J region (RBPJ) and activate transcription [7].

Because Notch signaling controls the homeostasis of different physiological processes, its alteration may cause different pathological states or diseases including inherited syndromes or acquired malignancies [9, 10]. Notch operates in the context of the cellular microenvironment and the immune system [11, 12] to process either proliferative, pro-differentiation, or quiescence/stem cell maintenance signals [13–15]. Thus, it does not surprise that, depending on the cellular events, Notch may act as an oncogene or a tumor suppressor [9].

The majority of oncogenic alterations of NOTCH receptors in human cancers occur in the *NOTCH1* gene. Activating *NOTCH1* mutations are prevalent in hematological malignancies and sequenced in 40% to 70% of T cell acute lymphoblastic leukemia (T-ALL) [16], in 10–15% of chronic lymphocytic leukemia (CLL) and mantle cell lymphoma (MCL) [17, 18] and a subset of diffuse large B cell lymphoma (DLBCL) [19]. These mutations have been also described in solid cancers, such as breast cancer [20, 21], medulloblastoma [22], lung adenocarcinoma [23], melanoma [24], and colon cancer [25]. Most of these mutations occur in the juxta-membrane heterodimerization (HD) domain, which holds together the NECD with NTM, or in the PEST (rich in proline (P), glutamic acid (E), serine (S), and threonine (T)) degron domain, and result in a more stable and transcriptionally active NICD [16]. On the opposite, loss-of-function mutations occurring in the NECD module are detected in the skin squamous cell carcinoma (SCC) [26, 27], head and neck cancer [28, 29], and myeloid leukemia [30, 31].

Notch controls both cell-intrinsic and extrinsic circuits leading to tumor development, progression, and response to therapy. Several therapeutic efforts have historically focused on modulating Notch signaling by using small molecules such as γ-secretase inhibitors (GSI) or antibody-based strategies, albeit without achieving clinical translation [32, 33]. These approaches have a poor therapeutic window—wild-type versus mutant proteins—limiting their application in human diseases [34]. However, this is not the case for small molecules targeting SERCA. SERCA inhibition hijacks Notch1 trafficking and its activation emerging as a druggable approach for *NOTCH1*-dependent cancers [35]. Here we update on the current scientific advancements to impede NOTCH1 transfer to the cell surface by blocking SERCA activity as a strategy to target *NOTCH1*-mutated cancers.

## SERCA

SERCA proteins belong to the superfamily of active transporters known as P-type ATPases (E_1_/E_2_-type) located in the ER. In 1993 Toyoshima and colleagues described the first complete structure of SERCA by cryo-electron microscopy [36]. Subsequently, novel high-content techniques shaped the resolution of several crystallography structures of SERCA [37]. These studies showed how ligands (e.g., vanadate, thapsigargin) bind SERCA, and what structural changes occur during the enzymatic catalytic cycle [38–42]. *ATP2A1* (16p11.2), *ATP2A2* (12q24.11), and *ATP2A3* (17p13.2) genes encode for SERCA1, 2, and 3, respectively [43, 44]. Today, over 70 SERCA isoforms resulting from alternative splicing are deposited in the Protein Data Bank database [45]. While these transcripts share up to 85% of sequence homology, differences in tissue distribution, Ca^2+^ binding affinity in both normal and cancer tissue, are due to changes in the protein C-terminal region [46–51].

**Fig. 1:**
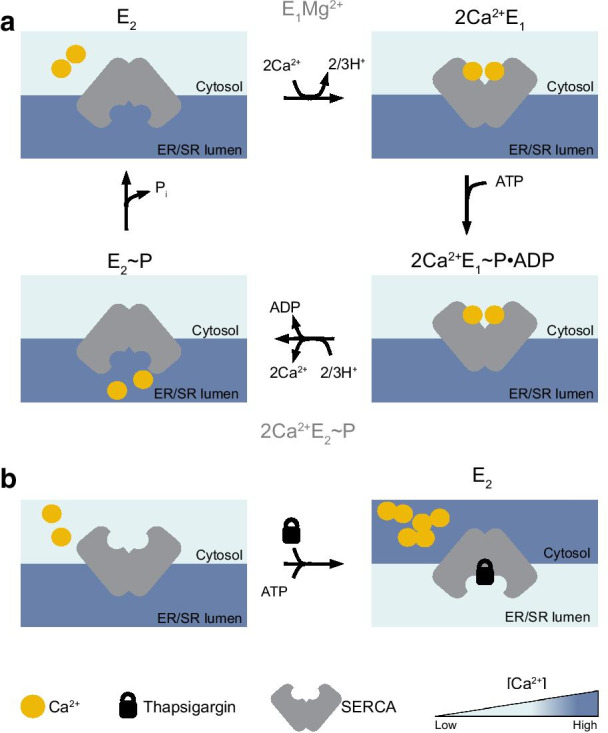
SERCA catalytic cycle. **a** SERCA pumps Ca^2+^ from the cytoplasm to the endoplasmic reticulum (ER) to create an ion gradient of ~ 10,000-fold across the cell membranes. During the catalytic cycle the Ca^2+^-ATPase switches between diverse oriented structures (E_1_ and E_2_) characterized by a different affinity for Ca^2+^ ions. The enzymatic transition between conformations catalyzes ATP in a stoichiometric ratio with Ca^2+^ of 1:2. E_1_ binds two molecules of cytoplasmic Ca^2+^ (2Ca^2+^E_1_, top right) and hydrolyzes one molecule of ATP to induce the high energy state of SERCA (E_1_ ~ P). Following the decay in ADP, E_2_ ~ P releases Ca^2+^ in the ER lumen. The cycle is closed and reopened along with the dephosphorylation of SERCA. **b** Thapsigargin (or other SERCA inhibitors) locks SERCA in the ground E_2_ state (“dead-end”) preventing Ca^2+^ binding and ATP hydrolysis. Thapsigargin blockade causes a continuum leakage of Ca^2+^ from the ER to the cytosol reverting the physiological polarization of Ca^2+^ across these two compartments

SERCA proteins maintain intracellular Ca^2+^ homeostasis by pumping Ca^2+^ from cytosol into the ER [52]. These 110 KDa pumps are organized in 10 transmembrane (TM) helices (M1-M10) along with two Ca^2+^ binding site (site I and II), a small luminal tail, three cytoplasmic domains (A, actuator; N, nucleotide binding; P, phosphorylation). These functional modules mediate ATP hydrolysis, hydron (H^+^), and calcium (Ca^2+^) binding and shuffling through the ER membrane [53]. Conformational changes during the catalytic cycle involving the N, A, and P domains along with the TM helices allow for a continued alternated access of Ca^2+^ from site I and II to the cytoplasmic and luminal sides [52]. The SERCA transport cycle is like the one described by Albers for other P-type ATPases [54] (Fig. 1a). The enzymatic reaction (E) alternates phases with high (2Ca^2+^E_1_) or low (E_2_) affinity to Ca^2+^ coupled with high (2Ca^2+^E_1_ ~ P) or low (E_2_ ~ P) energy phosphorylated states in the following sequence: E_2_ → 2Ca^2+^E_1_ → 2Ca^2+^E_1_ ~ P → 2Ca^+^E_2_ ~ P → E_2_ ~ P → E_2_. At the ground state (E_2_), SERCA presents two Ca^2+^ binding sites exposed to the luminal ER side [55]. In this orientation, ATP is located on the N domain far from the Asp^351^ residue of the P domain [56]. The exchange of 2/3 H^+^ causes a conformational change in the M1-M4 helices that enables a rotation of the A domain and the binding of cytosolic Ca^2+^ (2Ca^2+^E_1_). Next, the N domain shifts close to the Asp^351^ residue leading an energy-phosphorylated states 2Ca^2+^E_1_ ~ P [57, 58]. The following decay to a lower energy state, 2Ca^2+^E_2_ ~ P, sees a rotation of the N domain and A domain of 30° and 110°, respectively. Now the M1-M2 and M3-M4 helices pairs with the M5-M10 complex to release Ca^2+^ sites toward the luminal ER side [59, 60] in exchange of protons transferred to the cytosol. Dephosphorylation with the release of free phosphate, closes and reopens the cycle (E_2_) [61]. Thapsigargin and other SERCA inhibitors inhibit the enzyme in a Ca^2+^-free state (E_2_), avoiding the high-affinity binding of Ca^2+^ and the following activation of the catalytic cycle (Fig. 1b) [62–64].

## SERCA, disease, and cancer

The specific tissue distribution of SERCA isoforms accounts for their non-redundant role in physiological processes and human diseases [65]. For example, while the fetal (1b, 1001 amino acid -AA-) and adult (1a, 994 AA) SERCA1 isoforms are functionally identical, the difference in 7 AA may explain the contribution of SERCA1b in muscle development [66]. Similarly, SERCA2 transcripts encode for four isoforms (a-d) [61] with the highest affinity for Ca^2+^ (K_Ca_^2+^ 0.2 μM) [67] compared to SERCA1 and 3. The “a” isoform (997 AA) is expressed in slow-twitch skeletal and cardiac muscle, while the “b” (1042 AA) isoform is prevalent across different tissue [68]. The difference in size between the two variants is due to the presence in SERCA2b of an 11th transmembrane helix (TM11) and a luminal extension (LE) peptide which extends the C-terminal tail into the ER lumen [69, 70]. Biochemical and structural studies demonstrated that the TM11 tail is responsible for Ca^2+^ affinity, pump turnover, and the interaction with the ER redox and chaperon proteins [71, 72]. SERCA2c instead is expressed in cardiac muscle [73] and, at a lower extent, in hematopoietic and epithelial cells [74], while SERCA2d in skeletal muscle [75]. SERCA3 (a-f) is expressed in non-muscle tissues at variable levels and is frequently expressed with SERCA2b [76]. Compared to SERCA1 and 2, SERCA3 has the lowest affinity to Ca^2+^, suggesting that this pump is only active in the presence of higher cytoplasmic Ca^2+^ concentration [67].

Given their role in controlling cellular Ca^2+^ homeostasis, SERCA proteins have been involved in several human diseases from inherited syndrome to heart failure [77]. Loss-of-function mutations of *ATP2A1* cause Brody myopathy, a rare autosomal dominant genetic condition, characterized by painless muscles cramping and stiffening after exercise or cold temperatures [78, 79]. Similarly, *ATP2A2* mutations account for the development of the Darier disease (*keratosis follicularis*), a severe skin disorder characterized by skin wart-like blemishes, due to loss of adhesion between epidermal cells [80, 81].

The potential involvement of SERCA in cancer progression has been an active area of investigation given its role in Ca^2+^ homeostasis and its effect on cell survival and ER stress pathway [46]. For example, SERCA2 overexpression protects from apoptosis [77], while aberrant SERCA3 expression co-occurs in differentiating cells [46]. Park and colleagues showed that the overexpression of SERCA2 and Bcl-2 is a consequence of the repositioning of the nuclear factor kappa B (NF-kB) secondary to calcium/calmodulin-dependent protein kinase 2 alpha (CaMK2α) activation in metabolic stress-resistant breast cancer cell lines MDA-MB-231 and MCF-7 [82, 83]. They next showed that combined treatment with thapsigargin, a general SERCA inhibitor, and 2-deoxy-d-glucose (2-DG, acting as an NF-kB inhibitor) reduces the tumor burden, while 2-DG alone, that mimics glucose starvation, had a lower effect in a breast cancer xenograft model [83]. Overall, these data suggest that SERCA2 overexpression is a general mechanism to evade apoptosis and may result from the activation of metabolic stress [84].

A second observation is that SERCA expression may vary during epithelial differentiation and carcinogenesis playing different roles depending on the tissue of origin. For example, in colorectal cancer (CRC) increased SERCA2 transcripts correlate with tumor node metastasis or higher tumor grade [85], while decreased *ATP2A2* mRNA abundance is measurable in transformed thyroid cells [86]. Similarly, SERCA3 protein levels seem to correlate with the grade of epithelial differentiation. In CRC, SERCA3 expression increases in colonic mucosa and hyperplastic polyps, while it reduces in dysplastic, moderately, or poorly differentiated carcinoma cells [87]. In lung adenocarcinoma SERCA3 mRNA is reduced compared to non-transformed cells, but after pharmacologic-induced differentiation, the physiologic expression levels of the enzyme are restored [88].

Interestingly, the expression of SERCA isoforms is not mutually exclusive in cancer cells. Cancer cells may simultaneously have multiple SERCA isoforms, and the net contribution to the cancer phenotype is a balance between the expression of different transcripts [89]. For example, SERCA2 and SERCA3 are oppositely regulated in acute promyelocytic leukemia (APL) cell lines HL-60 and NB4 and freshly isolated APL cells treated with the pro-differentiating agent all-trans-retinoic acid (ATRA) in vitro [90]. A similar effect is seen during the differentiation of MEG 01, UT7, M-07e, and CHRF 288-11 erythro-megakaryoblastic leukemia cell lines treated with 10^–8^ M of the phorbol ester PMA [91]. Collectively these studies suggest that the downregulation of SERCA3 and overexpression of SERCA2 are key processes in leukemia stem cell and cancer maintenance [46].

While SERCA mutations are rare in cancer, some studies have demonstrated the involvement of genetic alterations of *ATP2A* genes in tumor development, mainly for lung and colon cancer [92]. Liu and colleagues showed that heterozygous mutant *ATP2A2* mice develop late-onset squamous cell tumors in the gut and in the skin where the expression of SERCA2 protein is reduced due to haploinsufficient loss-of-function mutations [93]. Toki and colleagues confirmed these initial observations and showed that the onset of *ATP2A2*-deficient tumor depends on the level of SERCA residual activity [94], suggesting that SERCA haploinsufficiency may predispose to multistage carcinogenesis by altering Ca^2+^ homeostasis [95].

## SERCA and Notch

In 1999, Goran Periz and Mark E. Fortini described that the trafficking events leading to a correct NOTCH activation may be disrupted in the presence of a defective Ca^2+^-ATPase function in a *Drosophila* model [96]. In this work, the authors demonstrated that loss-of-function alleles of the *Drosophila* SERCA homologous gene *Ca-P60A* alter proper synthesis, folding, and trafficking of the NOTCH receptor in the ER/Golgi compartments. Consistently, in *Drosophila* S2 cultured lines, the treatment with general SERCA inhibitors such as thapsigargin and cyclopiazonic acid (CPA) primarily reduces the amount of NOTCH proteins that reach the cell surface [96]. While extremely interesting, these observations were not confirmed in mammalian cells until the Stegmaier’s laboratory embarked on a large gene expression-based screening (GE-HTS) effort to identify inhibitors of oncogenic *NOTCH1* signatures or enhancer of *NOTCH1* HD mutant L1601P∆P activity in T-ALL. Among the top hits were the genes *ATP2A2* and *ATP2A3*, and SERCA inhibitors such as thapsigaricin (an analog of thapsigargin) and CPA. Together with other ion flux modulators, SERCA emerged as a novel potential therapeutic target in *NOTCH1*-associated cancers [35, 97]. Furthermore, these data suggest the hypothesis that Notch signaling could be dysfunctional in several genetic disorders associated with loss of function *ATP2A1-3* mutations.

## Thapsigargin and derivatives

### Thapsigargin

The rise of SERCA inhibitors for cancer therapeutics dates back to 1960 when the National Cancer Institute (NCI) launched a program to identify compounds with antitumor activity from 35,000 plant extracts [98]. Sesquiterpene lactone (SL) derivatives demonstrated anti-inflammatory and antitumor activity in several tumor types, like laryngeal carcinomas, uveal melanomas, pituitary macroadenomas, kidney, prostate cancer, and hematological malignancies [99–104]. Among others, thapsigargin, parthenolide, and artemisinin were selected for their potency and initially used as tool compounds in different cancer models. Several SERCA inhibitors which differed in their source, chemical structure, potency and binding affinity to SERCA isoforms were subsequently developed [84].

Thapsigargin is an SL phyto-derivative compound isolated from the umbelliferous Mediterranean *Thapsia Garganica* [105]*.* Thapsigargin binds SERCA in its E_2_ Ca^2+^-free conformation through an irreversible lipophilic interaction, by stabilizing SERCA in a so-called dead-end inactive state with low Ca^2+^ affinity, preventing both ATPase and Ca^2+^ transport activity [42, 106]. Thapsigargin binds to all SERCA isoforms with different specificity within the transmembrane helices M3 (at Phe^256^), M5 (at Ile^765^), and M7 (at Tyr^837^) [42, 107]. SERCA1 appears to be the more sensitive to thapsigargin inhibition (K_i_ ~ 0.2 nM), while the affinity for SERCA2 (K_i_ ~ 1 nM) and SERCA3 (K_i_ ~ 12 nM) decreases of 20 and 60 times, respectively (Table 1) [50]. Following SERCA inhibition, the Ca^2+^ depletion from ER produces a modification in the plasma membrane permeability to extracellular Ca^2+^, leading to a rise of intracellular Ca^2+^ level that occurs as early as one-two minutes following thapsigargin treatment [103]. The initial increase of intracellular Ca^2+^ is followed by a second cytosolic peak in Ca^2+^ concentration that precedes apoptosis occurring within 24–48 h [103]. These on-target Ca^2+^ effects can be seen at sub-nanomolar range (K_i_: 10^–10^ M) in several cell types including normal tissue [108].

**Table 1:** SERCA inhibitors

Structure	Compound	Binding site	IC_50_ (μM)	Cell line/in vivo	References	Clinical trial
	Artemisinin	Leu^263^, Phe^264^, Gln^267^, Ile^977^, Ile^981^, Ala^985^, Asn^1039^, Leu^1040^, Ile^1041^ and Asn^1042^^(*)^	9.6	Breast cancer	[109]	N/A
			> 40	NSCLC	[110]	
			> 60	Prostate cancer	[111]	
			> 60	Renal cancer		
			> 60	Ovarian cancer		
			12–60	Leukemia		
			101–(> 1000)	Melanoma	[112]	
			> 50	Neuroblastoma		
			156–204	Colon cancer		
			31–(> 1000)	Pancreas cancer		
			9.7	HCC	[113]	
	CAD204520	Asp^59^ (M1), Val^62^ (M1), Asn^101^ (M2), Asp^254^ (M3), Pro^312^ (M4)	2.1–9.9	T-ALL	[114]	N/A
			1.4–12.7	MCL		
			N/A	T-ALL xenografted model		
	Casearin J	N/A	0.7–2.5	T-ALL	[115, 116]	N/A
	Curcumin	Hydrophobic task between M3 and M5	7–15	Purified SERCA	[117]	NCT02064673 NCT04403568 NCT04266275 NCT01490996 NCT00094445
			50	Glioblastoma	[118]	
			10–20	Colorectal cancer	[119]	
			10–20	B-ALL	[120]	
			15 ± 6.8–25 ± 5.2	Cervical cancer	[121]	
			11.31 ± 1.47	Breast cancer	[122]	
			5.5–11.6	HNSCC	[123]	
			N/A	Liposarcoma xenografted model	[124]	
	CXL017	N/A	1.04	NCI-60 cancer cell line panel	[125]	N/A
			13.5 ± 0.5	AML	[126]	
	Cyclopiazonic acid	Gln^56^ (M1), Asp^59^ (M1), Asn^101^ (M2), hydrophobic indole group (M3, M4)	0.175	AML	[127]	N/A
			0.125	Renal carcinoma		
			0.09 (SERCA1b) 2.5 (SERCA2b) 0.6 (SERCA3a)	Purified SERCA	[50]	
			14.76–17.84	T-ALL	[35]	
	DBHQ	Asp^59^ (M1), Pro^308^ (M4)	7 ± 4 (SERCA1b) 2.6 ± 1.3 (SERCA2b) 1.7 ± 1 (SERCA3a)	Purified SERCA	[50]	N/A
	sHA 14–1	N/A	29.2 ± 4.9 (SERCA1a) 23.5 ± 4.2 (SERCA2b)	Purified SERCA	[128]	N/A
			50	B-ALL		
	Thapsigargin	Phe^256^ (M3); Ile^765^ (M5); Tyr^837^ (M7)	2.1 × 10^–4^ (SERCA1b) 1.3 × 10^–3^ (SERCA2b) 0.012 (SERCA3a)	Purified SERCA	[50]	N/A
			0.007 ± 0.001–3 ± 1	AML	[126]	
			~4	Adrenocortical carcinoma (ACC)	[129]	
			N/A	ACC xenografted model		
			3.7 × 10^–4^-14.67	NSCLC	[35, 130]	
			0.13–3.94	AML		
			5.5 × 10^–4^-0.026	Breast cancer		
			0.021	Cervix cancer		
			1.7 × 10^–3^	Melanoma		
			1.8 × 10^–3^-0.038	T-ALL		
			7.8 × 10^–3^-0.011	Prostate cancer		
			N/A	*Drosophila* intestinal stem cell model		
			N/A	T-ALL xenografted model		

^(*)^The binding site of artemisinin is referred to PfATP6 on *Plasmodium Falciparum* based on computational analysis and docking simulations [131, 132]

**Fig. 2:**
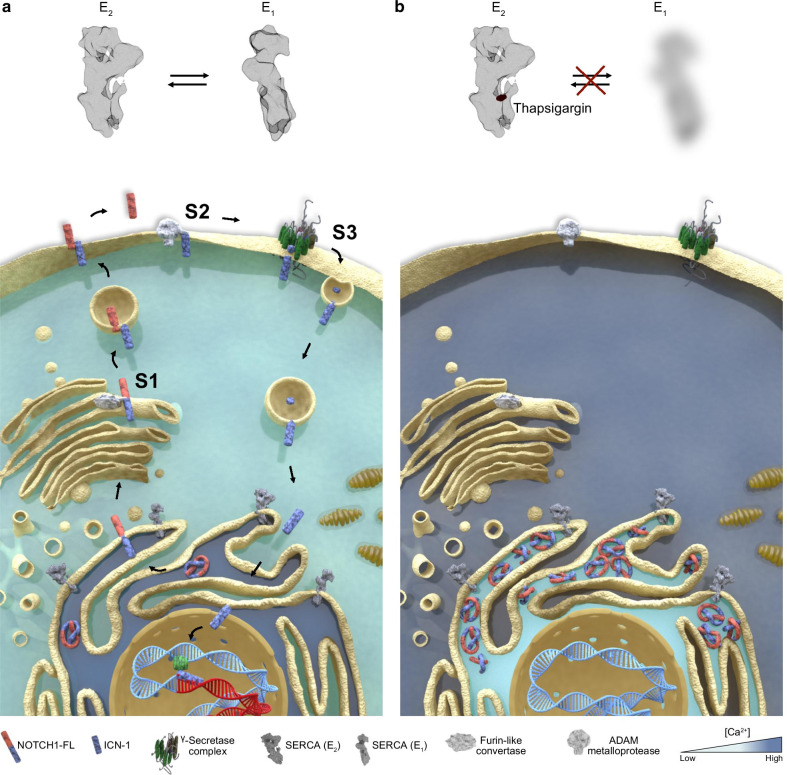
SERCA and the Notch trafficking. **a** Schematic representation of the effects of SERCA inhibition on the maturation and trafficking of NOTCH1. In physiologic conditions, SERCA pumps Ca^2+^ into the ER required for the proper folding of NOTCH1 proteins. A furin-like protease (S1) releases from the ER/Golgi the non-covalent heterodimer NFL1 that migrates through the cytosol toward the cell membrane. Following the interaction of the extracellular NECD1 with the Notch ligands, NOTCH1 is cleaved sequentially by metalloproteases (S2) and γ-secretases [GS (S3)]. The final cleaved protein NICD1 migrates to the nucleus to complex with co-activators and activates transcription. **b** SERCA blockade by SERCA inhibitors (e.g., thapsigargin) induces a leak of ER and the accumulation of the full-length isoform of NOTCH1 at the ER/Golgi level. As a consequence, no substrate for metalloprotease or γ-secretase is available with the result of a reduced level of nuclear NICD1 proteins

In the initial study in T-ALL, Roti and colleagues demonstrated that SERCA inhibition disrupts the trafficking of mutated NOTCH1 receptors that consequently accumulate in the ER/Golgi compartments upon drug treatment (Fig. 2a, b). This defective processing of newly synthesized NOTCH1 peptides ultimately results in a net reduction of NTM1 on the cell surface, the substrate of the γ-secretase complex. Hence, the consequent decrement on NICD1 level causes a Notch1 on-target inhibitory effect on leukemia growth in vitro, in T-ALL xenografts, and in a *Drosophila* intestinal stem cell model in which Notch1 inhibition perturbs differentiation of midgut pluripotent stem cells [35, 96]. Interestingly, mice treated with thapsigargin did not develop gastrointestinal toxicity, in sharp contrast with previous evidence of preclinical and clinical studies assessing the role of GSI [133]. These results suggested that mutated NOTCH1 receptors were more sensitive to the effects of thapsigargin than wild-type NOTCH1/NOTCH2 proteins expressed in normal cells providing a clinical therapeutic window for SERCA inhibitors.

Sharma and colleagues confirmed this original observation and demonstrated that thapsigargin alone or in combination with the monoclonal anti-NOTCH1 antibody mAb 604.107 inhibited “gain of function” mutants associated with T-ALL such as L1594P, R1599P, and I168N [134]. An important question is whether the lack of NOTCH1 Ca^2+^ binding modules EGF-like and LNR domains circumvents the requirement of the Ca^2+^-ATPase function. NOTCH1 peptides that are similar to the membrane-bound furin-processed forms ΔEGFΔLNR or NICD1 are insensitive to thapsigargin inhibition, and they properly localize to the cell surface, suggesting that the Ca^2+^-ATPase activity is required for the furin-mediated cleavage of NOTCH1 precursors in T-ALL cells [35]. These results differ from studies in flies where ΔECN (equivalent of ΔEGFΔLNR) proteins fail to correctly localize on apical eye disc membranes [96]. Collectively these data suggest that Ca^2+^ binding motives are required for thapsigargin effects and that Ca^2+^-ATPase activity interferes with general trafficking and secretion of NOTCH1. However, Roti and colleagues reported that ~ 25–30% of thapsigargin-treated mice (~ 0.4 mg/kg/injections/day) died during the study with no prior weight loss or clinical signs of illness, suggesting that at these doses thapsigargin may cause a sudden cardiac lethal event [35]. A different thapsigargin schedule, 0.4 mg/kg/injections 3 times per week, is tolerated and effective in reducing Notch1 signaling in TNF-induced synovial M1 macrophages in a Hes1-GFP/TNF transgenic mouse model of rheumatoid arthritis [135]. Nevertheless, despite the emerging role of SERCA as a Notch1 druggable target, the transition of naive thapsigargin into the clinic is worrisome because of the effect of this drug on cardiac SERCA2a [136].

### Mipsagargin and JQ-FT

**Fig. 3:**
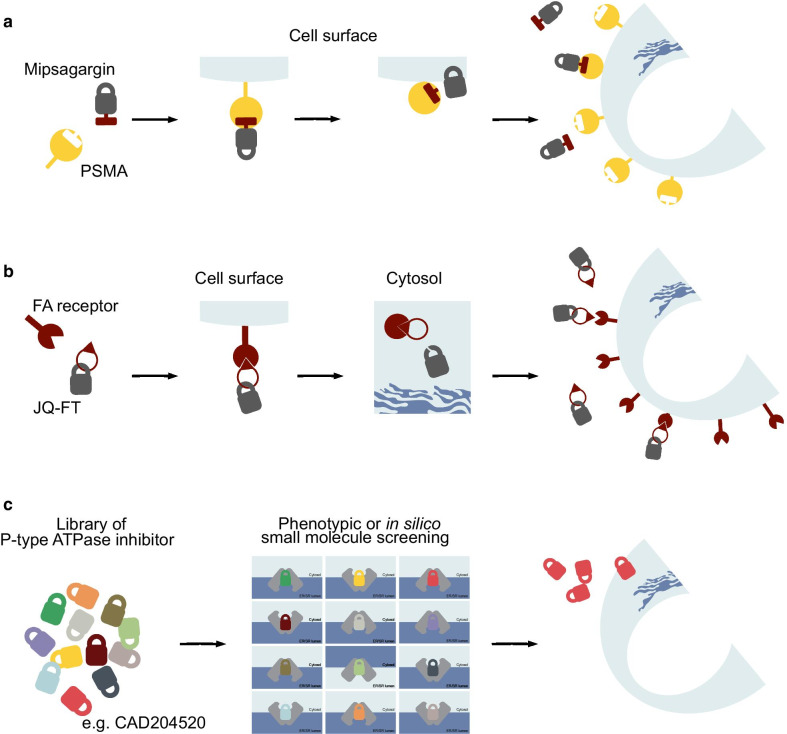
Strategy to overcome thapsigargin’s toxicity. **a** Mipsagargin is a thapsigargin derivative coupled with a masking peptide that is a substrate for the carboxypeptidase prostate-specific membrane antigen (PSMA). This peptide reduces the affinity of these molecules toward SERCA in non-neoplastic cells. However, if mipsagargin intercepts PSMA on tumor cells or neoplastic neoangiogenic vessels are cleaved into a cytotoxic analog of thapsigargin (12-ADT-Asp) and diffuse into the cancer cells. **b** JQ-FT is a derivative of thapsigargin, 8-*O*-debutanoyl-thapsigargin, linked with folic acid. In tumor cells expressing folate (FA) receptor, JQ-FT enters through endocytosis and proteases release 8-*O*-debutanoyl-thapsigargin from FA directly into the cytosol of targeted cells. **c** Identification of SERCA inhibitors by high-throughput screening, ATPase activity assays (Ca^2+^-ATPase vs. Na^+^/K^+^-ATPase vs. H^+^/K^+^-ATPase) or in silico prediction

Applying lessons from the experience of monoclonal antibody-based strategies in cancer, one way to overcome limitations associated with thapsigargin clinical translation is by developing pro-drugs that selectively target the desired cell type [103]. This is, for example, the mode of action of mipsagargin/G202, a thapsigargin derivative coupled with a peptide cleaved by the carboxypeptidase prostate-specific membrane antigen (PSMA) [137] (Fig. 3a, Table 2). PSMA is overexpressed in the neovasculature of several tumors including hepatocellular carcinoma, mesothelioma, ovarian, bladder, renal, and breast cancer [138]. In biochemical assays, G202 showed a lower SERCA inhibitory capacity and potency confirming previous studies that most of the derivatives [139] are less potent compared to thapsigargin, hence potentially less effective in vivo [140]. Nevertheless, three days of mipsagargin treatment at 56 mg/kg led to a reduction of more than 50% in tumor size in prostate cancer cell xenograft models expressing PSMA [137]. These proof of concept preclinical data justified phase II study clinical trial (NCT01777594) in patients with advanced sorafenib-refractory hepatocellular carcinoma (HCC) [141]. Patients treated with mipsagargin at 40 mg/m^2^ on days 1–3 (dose level -1) or 40 mg/m^2^ on day 1 and 66.8 mg/m^2^ on day 2–3 (dose level 1) experienced a disease stabilization in 63% of cases, a decrease in tumor blood flow, a median time to progression (TTP) of 134 days (*P* < 0.001), compared to the historic median TTP of 63 days, a progression-free survival (PFS) of 129 days, and overall survival (OS) of 205 days [141, 142]. Patients treated with mipsagargin at dose level -1 experienced grade 1 and 2 adverse events (AE) such as increased blood creatinine (68% of patients), fatigue (56%), and nausea (44%) [141]. These AE were seen also in patients with advanced/recurrent glioblastoma multiforme, where mipsagargin treatment led to disease stabilization in 22% of patients [143].

**Table 2:** Thapsigargin derivative compounds

Structure	Compound	Binding site	IC_50_ (μM)	Cell line/in vivo	References	Clinical trials
	Mipsagargin (G202)	M3, M4 (Gln^250^), M5, M7 A phospholipid head group from the ER membrane interacts with the free amino acid of 12ADTβAsp, near the binding pocket for CPA	10.964 ± 0.4	Bladder cancer (PSMA−)	[137]	NCT01056029 NCT02067156 NCT01777594 NCT02607553
			0.191 ± 0.029	Prostate cancer (PSMA+)		
			N/A	Prostate and breast cancer xenografted models		
	JQ-FT	N/A	1–10	T-ALL	[145]	N/A
			N/A	T-ALL xenografted model		

Instead of an enzymatic pro-drug-based approach such as the one described for prostate-specific antigen [144] and PSMA [137], Roti and Qi leveraged the dependency of ALL on folic acid (FA) metabolism. They first demonstrated that folate receptor 2 (FR2) is aberrantly expressed in T-ALL and that the endocytic trafficking of this receptor can be used as a carrier for folate-conjugated probes. They next showed that the alcohol derivative of thapsigargin suitable for conjugation, 8-*O*-debutanoyl-thapsigargin, similarly to the parental drug inhibits NOTCH1 and it is preferentially active against mutant T-ALL. They then connected the carboxylate of folic acid to the C8-alcohol of 8-*O*-debutanoyl-thapsigargin via a cleavable ester linkage to generate JQ-FT (Table 2) [145]. In a series of preclinical validation studies, the authors demonstrated that JQ-FT is stable in cell culture conditions and that the cleavage of JQ-FT occurs through an endocytic FR-mediated process (Fig. 3b). Moreover, JQ-FT is 150-fold more tolerable in mice compared to unconjugated thapsigargin, without killing the antileukemic effect in a preclinical *NOTCH1* mutated T-ALL model in vivo. This approach enhanced the therapeutic window of thapsigargin as a NOTCH1 inhibitor and provided dual selectivity: leukemia over the normal cell and *NOTCH1* mutated over wild-type receptors [145].

## Cyclopiazonic acid and 2,5-Di-(tert-butyl)-1,4-benzohydroquinone

An alternative strategy to reduce the potential toxicity of thapsigargin or to avoid complicated chemical synthesis routes is through the identification of SERCA inhibitors that retain the anti-Notch properties but lack Ca^2+^-related toxicities. For example, CPA and 2,5-Di-(tert-butyl)-1,4-benzohydroquinone (DBHQ) have been recognized for their ability to lock SERCA in a pocket different from the one of thapsigargin [41, 50, 146]. In fact, the CPA binding pocket is located between the SERCA transmembrane helices M1, M2, M3, and M4 helices, with polar interaction at the Gln^56^, Asp^59^, and Asn^101^ residues [41], while DBHQ at Asp^59^ on and Pro^308^ [146]. Both compounds, similar to thapsigargin, reversibly stabilize SERCA in the E_2_ ~ P conformational state (Table 1) [84, 146]. CPA and DBHQ are less potent compared to thapsigargin; in fact, CPA inhibits SERCA1 with a K_i_ is 120 nM while DBHQ with a K_i_ of 0,4 μM [50]. Because of their poor pharmacokinetic properties and lack of potency, DBHQ and CPA had a limited application in tumor models, with only few studies exploring the feasibility of their application as anticancer or antimalarial compounds [40, 50, 147–151]. However, similarly to thapsigargin, CPA induces a *NOTCH1* off transcriptional program and triggers a NOTCH1 trafficking defect, suggesting that a thapsigargin-like binding mode of action is not an absolute requirement to achieve the suppression of the Notch1 signaling [35].

## Emerging SERCA inhibitors

### Dual target compounds

Because P-type ATPases are compelling therapeutic targets in several human diseases [152], several efforts are ongoing to develop tolerable SERCA inhibitors for cancer therapeutics (Table 1). Works from Xing’s laboratory demonstrated that a stable analog of a putative Bcl-2 inhibitor HA 14-1, sHA 14-1, moderately inhibits SERCA1a and 2b with IC_50_ values of 29.2 ± 4.9 and 23.5 ± 4.2 μM, respectively [128, 153]. Mechanistically, sHA 14-1 induces a rapid ER Ca^2+^ release that triggered the expression of ER stress-associated transcription factor ATF4/CREB-2 and apoptosis in B cell acute lymphoblastic leukemia (B-ALL) cell lines [128]. The same group pursued a structure–activity relationship (SAR) optimization of sHA 14-1 that led to the identification of the ethyl 2-amino-6-(3,5-dimethoxyphenyl)-4-(2-ethoxy-2-oxoethyl)-4*H*-chromene-3-carboxylate (CXL017) [125, 154]. Similar to the parent compound, CXL017 simultaneously inhibits SERCA and Bcl-2 [155] while induces apoptosis in hematopoietic multi-drug-resistant cancer cell lines including T-ALL [126]. Additional studies demonstrated that the active isomer (−) CXL017 synergizes with other SERCA inhibitors including thapsigargin, CPA, and DBHQ, indicating that CXL017 may bind SERCA at a unique allosteric site [126].

### Casearin J

Using in silico approaches and docking simulation, De Ford and colleagues demonstrated that tricyclic clerodane diterpenes (TCD) may target SERCA [116]. In the following study, they showed that TCD isolated from *Casearia sylvestris* casearin J (CJ) [115], affects the Notch1 pathway in human T-ALL cells. CJ reduces the cell surface expression of NOTCH1 receptors and prevents the formation of the cleaved NICD1 molecules, which results in the transcriptional suppression of *MYC* and *HES1*. The authors also demonstrated that CJ is more active against HD-mutated T-ALL cells compared to a cell line carrying *NOTCH1* juxtamembrane mutations such as Jurkat. While this study does not rule out the activity of CJ on *NOTCH1* wild-type cells or mechanistically on NOTCH1 full length (NFL1) proteins, it confirms that T-ALL is sensitive to Ca^2+^-ATPase suppression further supporting the need to explore SERCA inhibitors with binding sites different from the one of thapsigargin in search of small molecules with tolerable off-target effects.

### Curcumin

Among other SERCA inhibitors, curcumin has been extensively tested in several health conditions due to its antioxidant, anti-inflammatory, and anticancer properties [156, 157]. Curcumin (1,7-bis(4-hydroxy-3-methoxyphenyl)-1,6-heptadiene-3,5-dione), also known as diferuloylmethane, is the main natural polyphenol extracted from the rhizome of *Curcuma longa* (turmeric) and in others *Curcuma spp*. [158]. Curcumin binds SERCA in a hydrophobic task between M3 and M5 [159] and stabilizes the enzyme in its 2Ca^2+^E_1_ conformational state preventing ATP binding [117]. Curcumin shows K_i_ values of 5.8 ± 1.6, 8.6 ± 2.5, and 53 ± 6 μM for SERCA1b, SERCA3a, and SERCA2b, respectively [50], although its poor bioavailability has been tested in vivo [124] and in clinical trials (NCT00094445, NCT01490996) [160, 161] without clear evidence of therapeutic benefits. Furthermore, curcumin (CU) was combined with standard chemotherapy [162], FOLFOX (5-fluorouracil, folinic acid, and oxaliplatin), in advanced metastatic CRC. CUFOX was well tolerated, and adverse events (AEs), quality of life, and neurotoxicity were comparable to those seen in standard chemotherapy [161]. While the study was too small (28 patients) and the cohorts potentially biased for tumor staging and number of metastatic sites, the authors reported a significant increase in OS and PFS in patients treated with CUFOX compared to FOLFOX alone [161].

Because curcumin has been shown to target multiple signaling molecules, several investigators pointed to the role of this drug in the suppression of the Notch signaling, albeit without a unifying mechanism. For example, Liu and colleagues showed that curcumin (10–90 μM) inhibits the proliferation of the SMMC-7721 hepatoma cancer cell line and suppresses *NOTCH1* mRNA and protein expression [163] in a way that was similarly described in osteosarcoma cells [164]. Subramanian and colleagues showed that 30 μM curcumin represses *NOTCH1, Jagged-1*, and *HES1* transcript and consequently diminishes NICD1 level in TE-7 esophageal cancer cells. The authors also demonstrated that curcumin reduces the transcription and the expression of proteins of the γ-secretase complex such as presenilin-1 and 2 (PSEN-1, 2), nicastrin (NCSTN), anterior pharynx-defective-1 (APH-1), and presenilin enhancer-2 (PEN-2). These results suggest a general mechanism of transcriptional inhibition or protein stability rather than a direct effect of curcumin on the γ-secretase complex as claimed in the manuscript [165]. An alternative hypothesis is that Notch1 signaling is decreased upon curcumin treatment as a consequence of the inhibition of a NOTCH1 transcriptional regulator [166]. Curcumin analogs with enhanced activity toward SERCA [85] or with a novel binding site between M3, M4 helices and the L78 loop [167] have been recently developed; however, their role as potential Notch modulator is far to be addressed [168].

### CAD204520

Despite the potential risk of Ca^2+^-related toxicity, SERCA inhibitors are used for clinical applications indicating that for some of these molecules the leak of ER Ca^2+^ toward the cytosol is moderate or compensated. This is probably the case for cisplatin, a widely used platinum-containing compound that among other effects inhibits SERCA and Na^+^/K^+^-ATPase simultaneously [169] or the SERCA antimalarial drug artemisinin and its derivatives (artesunate and di-hydro-artemisinin) that are tolerable with minimal side effects [170, 171]. As such the effect of a given SERCA inhibitor on cytosolic and ER Ca^2+^ levels may depend on its molecular mechanism of interaction with the ATPase [172].

From a secondary analysis of a small molecule screening of 191,000 compounds, Marchesini and Gherli identified one of such molecules (Fig. 3c) [114], CAD204520, with favorable pharmacodynamic properties. CAD204520, (4-[2-[2-[3-propyl-6-(trifluoromethoxy)-1H-indol-2-yl]-1-piperidyl]ethyl] morpholine) dihydrochloride, binds SERCA between the transmembrane helices M1, M2, M3, and M4 with two polar interactions to Asp^59^ on M1 (2.9 Å) and Asn^101^ on M2 (2.7 Å) and with several hydrophobic interactions involving Leu^61^, Val^62^, Ile^307^, Pro^308^, and Pro^312^. This binding groove is similar to the binding of CPA and DBHQ but different from that of thapsigargin. Interestingly, in preclinical studies, CAD204520 suppresses mutated Notch1 signaling without causing overt cardiac toxicity. We demonstrated that in cardiomyocytes isolated from Wistar rats CAD204520 treatment reduces contractile efficiency by ~ 25%, a cardiomechanics impairment that is tolerated at a therapeutic concentration in a T-ALL orthotope model in vivo. These results are consistent with the transient effects on cytosolic Ca^2+^ shifts and the lack of unfolded protein response (UPR) activation in cardiomyocytes upon CAD204520 treatment. Collectively these data also support the work from Sehgal and colleagues that showed that inhibition of SERCA ATPase activity and apoptosis can be efficiently achieved without triggering measurable changes in Ca^2+^ pools [172]. This effect is a consequence of how ligands lock the Ca^2+^-ATPase, kinetics, and the number of rotating bonds, suggesting that the development of new SERCA inhibitors requires careful consideration of substrate binding [173]. A second question concerning how SERCA inhibitors bind to the Ca^2+^-ATPase is whether different binding sites may cause a different mechanism of resistance. Our team and others have demonstrated the rapid cell adaptation to thapsigargin treatment due to mutations occurring in the M3 segment spanning between Asp^254^ and Leu^260^ [114, 174, 175]. However, in these cells, we showed the lack of cross-resistance with CAD204520, suggesting that early characterization of the binding mechanism may overcome potential relapse from mutant clones.

## Conclusions

Modulation of intracellular Ca^2+^ homeostasis plays critical roles in key processes that regulate cellular survival, growth, differentiation, metabolism, and death in normal and cancer cells. Thus, it is not surprising that several anticancer agents suppress pro-survival and activate pro-apoptotic pathways through modulation of Ca^2+^ signaling-dependent mechanisms. This is, for example, the case for chemotherapeutics such as cytotoxic alkylating agents [169] or antimetabolites that rely on a Ca^2+^ signaling component to induce cancer cell death [176]. Similarly, natural compounds including alkaloids, flavonoids, diterpenoids, and polyphenolics have been extensively investigated for their ability to modulate intracellular Ca^2+^ concentration and to participate in apoptotic signaling pathways. Among them, SL, such as thapsigargin, has been long regarded as target compounds for drug development. Thapsigargin has a broad spectrum of growth-suppressing activity in several tumor types including poorly dividing cells [177]. However, we have demonstrated the SERCA inhibition may efficiently control the trafficking of NOTCH1 and that this blockade can be achieved without causing overt cardiac toxicities in preclinical leukemia models [114]. Importantly the effects of SERCA suppression can be rescued by the overexpression of unprocessed NOTCH1 peptides such as NICD1, indicating that the antileukemia effect is on target for Notch1 inhibition rather than Ca^2+^ overload [35]. An important standing question is why mutated *NOTCH1* appears more sensitive to SERCA suppression compared to wild-type isoform or other proteins more broadly. One hypothesis to explain NOTCH1 and SERCA functional dependency is by mechanisms of co-regulation. It has been previously shown that PSEN and SERCA co-localize in the ER [178]. Since PSEN-1 is a key regulator of NOTCH1 maturation and preferentially binds NFL1 polypeptides processed through the ER, it is possible that NOTCH1-PSEN-1-SERCA is part of a co-functional protein complex. Interestingly, treatment of T-ALL cell lines with the selective PSEN-1 inhibitor MRK-560 inhibited mutant NOTCH1 processing and led to cell cycle arrest. MRK-560 treatment decreases leukemia burden and increased OS with no associated gut toxicity in T-ALL patient-derived xenografts in vivo, suggesting that, similar to SERCA inhibition, disruption of PSEN-1 may preferentially affect mutated proteins [179]. The second hypothesis is a Ca^2+^ mediated one. In fact, Malecki and colleagues previously demonstrated that clinically relevant activating *NOTCH1* HD mutations destabilize the NOTCH1-negative regulatory region and have deleterious effects on NOTCH1 folding and maturation. Because EGF and LNR repeats of NOTCH1 rely on Ca^2+^ for folding and activation, it may be possible that changes in ER Ca^2+^ may preferentially impair unstable NOTCH1 mutant proteins [180] compared to wild-type providing a therapeutic window for SERCA inhibitors. Finally, a hypothesis not yet explored to explain NFL1 accumulation at concentrations not sufficient to trigger the general mechanism of UPR is through a Ca^2+^-mediated transcriptional activation of inhibitors of furin-like proteases. This would explain for example why CAD204520 efficiently targets cancers with isolated PEST deletions that would not be predicted to be unstable given a normal LNR and HD protein sequence.

In conclusion, the transient disruption of SERCA activity can be leveraged for targeting Notch1 in cancers. Legitimate concerns associated with SERCA inhibition can be overcome by the identification of druggable modalities to lock ATPase function without causing dose-limiting ER Ca^2+^ toxicities.

## Data Availability

Not applicable.

## Abbreviations

2-DG: 2-Deoxy-d-glucose
AA: Amino acid
Adomain: Actuator domain
AE: Adverse event
APL: Acute promyelocytic leukemia
APH: Anterior-pharynx-defective
ATRA: All-trans-retinoic acid
B-ALL: B cell acute lymphoblastic leukemia
Ca^2^^+^: Calcium
CAD204520: (4-[2-[2-[3-Propyl-6-(trifluoromethoxy)-1H-indol-2-yl]-1-piperidyl]ethyl] morpholine) dihydrochloride
CaMK2α: Calcium/calmodulin-dependent protein kinase 2 alpha
CJ: Casearin J
CLL: Chronic lymphocytic leukemia
CPA: Cyclopiazonic acid
CRC: Colorectal cancer
CU: Curcumin
CXL017: Ethyl 2-Amino-6-(3,5-dimethoxyphenyl)-4-(2-ethoxy-2-oxoethyl)-4H- chromene-3-carboxylate
DBHQ: 2,5-Di-(tert-butyl)-1,4-benzohydroquinone
DLBCL: Diffuse large B cell lymphoma
DLL: Delta-like ligand
EGF: Epidermal growth factor
E: Enzymatic reaction
EFS: Event-free survival
ER: Endoplasmic reticulum
FA: Folic acid
FR: Folate receptor
GE-HTS: Gene expression-based high-throughput screening
GSI: γ-Secretase inhibitors
H^+^: Hydron
HD: Heterodimerization domain
HCC: Hepatocellular carcinoma
JAG: Jagged ligand
K_i_: Constant of inhibition
LNR: LIN-12/NOTCH repeats
MAML: Mastermind-like
MCL: Mantle cell lymphoma
MRD: Minimal residual disease
N domain: Nucleotide binding domain
NCI: National Cancer Institute
NCSTN: Nicastrin
NECD: NOTCH extracellular domain
NICD: NOTCH intracellular domain
NF-kB: Nuclear factor kappa B
NFL: NOTCH full length
NTM: NOTCH transmembrane domain
OS: Overall survival
P domain: Phosphorylation domain
PEN: Presenilin enhancer
PEST: Proline, glutamic acid, serine, threonine
PFS: Progression-free survival
PSEN: Presenilin
PSMA: Prostate-specific membrane antigen
RBPJ: Recombination signal binding protein for immunoglobulin kappa J region
SAR: Structure–activity relationship
SERCA: Sarco-endoplasmic reticulum Ca^2^^+^-ATPase
sHA 14–1: Stable analog of HA 14–1
SL: Sesquiterpene lactone
T-ALL: T cell acute lymphoblastic leukemia
TCD: Tricyclic clerodane diterpenes
TM: Transmembrane
TTP: Time to progression
UPR: Unfolded protein responses
